# Atrial Functional Mitral Regurgitation: From Diagnosis to Current Interventional Therapies

**DOI:** 10.3390/jcm13175035

**Published:** 2024-08-25

**Authors:** Panagiotis Iliakis, Kyriakos Dimitriadis, Nikolaos Pyrpyris, Eirini Beneki, Panagiotis Theofilis, Panagiotis Tsioufis, Vasileios Kamperidis, Konstantinos Aznaouridis, Konstantina Aggeli, Konstantinos Tsioufis

**Affiliations:** 1 First Department of Cardiology, School of Medicine, National and Kapodistrian University of Athens, Hippokration General Hospital, 115 27 Athens, Greece; panayiotisiliakis@gmail.com (P.I.); npyrpyris@gmail.com (N.P.); e.beneki@hotmail.com (E.B.); panos.theofilis@hotmail.com (P.T.); conazna@yahoo.com (K.A.); ntina.aggeli@gmail.com (K.A.); ktsioufis@gmail.com (K.T.); 2 First Cardiology Department, Medical School, Aristotle University of Thessaloniki, AHEPA University Hospital, 544 53 Thessaloniki, Greece; vkamperidis@yahoo.co.uk

**Keywords:** mitral regurgitation, atrial fibrillation, heart failure, atrial functional mitral regurgitation, mitral valve surgery, transcatheter edge-to-edge repair

## Abstract

Mitral regurgitation (MR) is one of the most common valvular pathologies worldwide, contributing to the morbidity and mortality of several cardiovascular pathologies, including heart failure (HF). Novel transcatheter treatment for MR has given the opportunity for a safe and feasible alternative, to surgery, in order to repair the valve and improve patient outcomes. However, after the results of early transcatheter edge-to-edge repair (TEER) trials, it has become evident that subcategorizing the mitral regurgitation etiology and the left ventricular function, in patients due to undergo TEER, is of the essence, in order to predict responsiveness to treatment and select the most appropriate patient phenotype. Thus, a novel MR phenotype, atrial functional MR (AFMR), has been recently recognized as a distinct pathophysiological entity, where the etiology of the regurgitation is secondary to annular dilatation, in a diseased left atrium, with preserved left ventricular function. Recent studies have evaluated and compared the outcomes of TEER in AFMR with ventricular functional MR (VFMR), with the results favoring the AFMR. In specific, TEER in this patient substrate has better echocardiographic and long-term outcomes. Thus, our review will provide a comprehensive pathogenesis and mechanistic overview of AFMR, insights into the echocardiographic approach of such patients and pre-procedural planning, discuss the most recent clinical trials and their implications for future treatment directions, as well as highlight future frontiers of research in the setting of TEER and transcatheter mitral valve replacement (TMVR) in AFMR patients.

## 1. Introduction

Functional or secondary mitral regurgitation (FMR) is defined as mitral regurgitation (MR) and is mainly attributed to functional abnormalities of left heart chambers [1]. The key pathophysiology basis of secondary MR is a disturbance in the balance between the closure forces and the tethering of mitral leaflets, which leads to poor or incomplete coaptation [2]. The two main morphological forms of FMR are atrial and ventricular FMR. In left ventricular functional MR (VFMR), the left ventricle (LV) undergoes regional or global remodeling and dysfunction, resulting in apical tethering of the mitral leaflets. It has been associated with numerous diseases (ischemic or not) affecting left ventricular myocytes [3]. On the other hand, atrial functional mitral regurgitation (AFMR) refers to the less common condition, in which MR, caused by annular enlargement, is associated with remarkable dilatation of the left atrium (LA) while both LV size and function are preserved [4]. The differentiation of patients enrolled and selected in studies, trials, cohorts, and registries, the multiple pathophysiological pathways involved (regarding both ischemic and non-ischemic heart disease), and the recognition of MR severity, as well as the lack of longitudinal data, make it difficult to address the true epidemiological data regarding secondary MR, and even more for AFMR [4,5]. AFMR is associated with atrial fibrillation (AF) [6], while sinus rhythm cardioversion seems to be associated with an improvement in MR severity [7], or heart failure with preserved ejection fraction (HFpEF) of the LV [8]. The left atrial overload in the aforementioned setting plays an important role in promoting the pathogenesis of AFMR. According to Carpentier’s classification, AFMR includes both type I (annulus enlargement) and type IIIb (restriction of the posterior leaflet) [9]. Nevertheless, the usual presentation is central MR, particularly in the early stages of left atrial dilatation [10], although AFMR may also present with posterior leaflet tethering, anterior leaflet pseudo-prolapse, and severe eccentric jet in the advanced stage [11]. The aim of this review is to discuss the pathophysiology and imaging assessment of AFMR and provide the most recent evidence with recommendations for surgical treatment and transcatheter edge-to-edge repair of AFMR.

## 2. Pathophysiology and Epidemiology of AFMR

### 2.1. Epidemiology and Prognostic Evidence

MR is one of the most common valvulopathies worldwide. It has been reported that approximately 2% of the global population suffers from MR, with rates expected to increase in the following years and reach up to 5 million people in America by 2030 [12,13]. As mentioned, regarding FMR, there are two distinct phenotypes, each with a distinct pathophysiological background, that formulate the spectrum of its diagnosis. This observation has become evident, as some patients with FMR notably did not have significant left ventricular (LV) dysfunction, while their MR was related to mitral annulus dilatation secondary to atrial fibrillation (AF), which improved after sinus rhythm restoration. Therefore, there have been several efforts to evaluate the specific epidemiology of AFMR and VFMR. The reported rates of AFMR in single-center, small cohort studies evaluating patients with MR or AF and preserved EF showed an AFMR rate between 8 and 27%, with increased rates in those with longstanding AF [14,15]. More recently, an analysis of the National Echo Database of Australia provided a large-scale estimation of AFMR in patients with an echocardiographic FMR diagnosis. In specific, this study, comprising approximately 14.000 patients, identified that AFMR is evident in 40% and VFMR in 60% of FMR diagnoses, showcasing that the true prevalence of AFMR might be larger than originally thought [16]. These findings are consistent with smaller cohorts, which showed a prevalence of 32–41% among FMR patients [15,17].

Along with examining the prevalence of this phenotype, investigators also questioned whether there was a prognosis difference in those individuals. The Australian Database study [16] showed that, following age, sex, AF, and pulmonary hypertension adjustment, AFMR had a significantly better prognosis than VFMR. At a median of 65 months follow-up, cardiovascular death occurred in 51% of AFMR and 57% of VFMR, while cumulative 5-year all-cause and cardiovascular mortality were significantly higher in VFMR compared with AFMR (62% vs. 50% and 49% vs. 25%, *p* < 0.001). Similar results have been shown by Dziadzko et al., which highlight the increased mortality of both VFMR and AFMR in comparison to the general population, but mention significantly higher mortality and HF rates [15]. Also, compared to degenerative MR, AFMR had worse survival and increased HF hospitalizations (*p* = 0.009 and *p* = 0.002, respectively), while these patients were less likely to undergo mitral interventions [18] Despite the increased events compared to non-FMR patients, it is important to highlight that in patients with mild–moderate AFMR, regression of the pathology is more common than progression (1.9 per 100 person-years vs. 3.9 per 100 person-years), while progression was only associated at a univariate level with mortality events. Other factors, non-AFMR associated, such as older age, lung disease, ischemic stroke, LV filling, and right ventricle systolic pressure were associated with mortality in this cohort. Interestingly, AFMR proregression was associated with a female-gender-increased LA volume index and a decreased LA ejection fraction (in the absence of AF) [19]. Future studies should focus more on identifying predictors of severe AFMR development and further investigate this relationship with excess morbidity and mortality.

### 2.2. Pathophysiology of AFMR

As mentioned, AFMR is mostly related to left atrial (LA) and mitral annular changes that are promoted by the presence of AF. In order to better understand the pathological changes in the mitral valve apparatus that result in MR, a brief description of key mitral anatomical and dynamic characteristics will be given. The mitral annulus is a hyperbolic paraboloid (saddle-shaped) structure anatomically separating the left atrium and ventricle. It is divided into an anterior and posterior part, with the anterior part coupled with the aortic annulus and the posterior part independently related to the LV inflow and the left atrial regions [20]. The distinctive saddle shape of this landmark is related to less mitral valve leaflet stress during cardiac systole [21]. It is mostly composed of fibrous tissue, thus limiting its ability to actively contract. Thus, the annulus shows three distinctive passive types of motion: annular translation, annular contraction, and annular folding, which are facilitated by both ventricular and atrial contraction, in systole and diastole, respectively [20]. Atrial contraction influences the proportion of the annular translation during end-diastole, as contraction of atrial annular fibers attracts the annulus away from the LV apex, thus facilitating a further increase in LV volume. In annular contraction, which happens during atrial and ventricular systole, the role of the atrium has been questioned. However, several investigators report that pre-systolic contraction accounts for more than 50% of the decrease in annulus area [22,23], and is based on an inwards contraction of the mitral apparatus due to LA circumferential fibers that passively contract the inner part of the annulus during late diastole, followed by a similar inwards contraction of the outer annulus in early systole due to LV fiber contraction [24]. Annular folding may also have an atrial element contributing to it; however, the specific mechanics are not elaborately understood. By these motions, the mitral annulus assists in mitral leaflet coaptation and in reducing mitral valve closing forces [21].

It is important to note that atrioventricular coupling is essential for the normal function of the annulus and the proper coaptation of the mitral valve leaflets. AF predominantly affects the capability of the atrium to contract, resulting in a loss of the “atrial kick” and therefore leading to ventricular-based annular mechanics. The loss of the atrial contractive contribution during diastole has been postulated as one of the culprits of AFMR pathogenesis and progression, promoting leaflet malcoaptation and delayed coaptation. In particular, the loss of annular size reduction during end-diastole results in a larger annular area, which enhances the annulus-leaflet imbalance, thus resulting in blood volume regurgitation.

Echocardiography studies comparing AFMR with AF control individuals have linked the presence of reduced mitral annular area fractional change with the presence of regurgitation [25,26,27]. More recently, in individuals with AF, Deferm et al. showed that mitral annular dynamics are decreased in persistent AF compared to sinus rhythm, significantly related to the limitation of the pre-systolic annular contraction, which is present in sinus rhythm but absent in AF. Differences in annular folding between the two groups were also noted, but did not reach statistical significance [28]. Interestingly, after sinus rhythm restoration, there were marked improvements in MR severity. Reduced mitral annulus contractility in AFMR patients has been also noted by Bai et al. [29], who, however, provided evidence regarding flattening of the saddle-shaped apparatus, as well as by Cong et al. [30] who showed that a reduced annular height to commissural width ratio, indicating saddle flattening, is an independent predictor of MR severity in AF. These results show that in the context of AF and absence of the atrial element of annular motion, the reduction in annular movement and the alteration of annular folding increase, as mentioned, the imbalance of annulus area and leaflet coaptation, as well as the stress applied to the leaflets during systole, resulting in MR.

Another pathophysiological implication in patients with AF and MR is annular dilatation, secondary to atrial dilatation. It is well known that structural atrial changes in individuals with AF result in dilatation of the LA [31]. This results in annular dilatation, which has already been implied, increases the mitral valve area and, when pathologically expanded, halts the physiological coaptation of the mitral leaflets. However, it has been demonstrated that the mitral leaflets are not static, but rather have the ability to increase in length in animal models of functional MR, linked to endothelial–mesenchymal transition [32]. However, clinical studies show that in AF patients ultimately developing MR, insufficient remodeling is related to disease progression. In more detail, Kim et al. increased mitral leaflet area in AF patients, compared to controls, in compensation to annular dilatation; however, this adaptation was the lowest in patients that developed MR, showing that not only annular dilatation, but also plateau of compensatory mechanisms lead to regurgitation [33]. Further supporting this mechanism, Kagiyama et al. reported that in AFMR patients, the annulus area was not an independent predictor of MR, although a small ratio of total leaflet area/annulus area was and indicated an insufficient leaflet remodeling response [11]. Despite the significance of these findings, there is still a gap in evidence regarding predictors or markers of sufficient leaflet remodeling response, which needs to be further analyzed in future trials.

Local anatomic relationships between the LA, LV, posterior annulus/leaflet, and AFMR-associated annular dilatation may further explain MR development. As first proposed by Silbiger [34], with respect to this LA enlargement, and as the posterior annulus, contrary to the well-anchored anterior annulus, anatomically independently separates internally the LA margin from the external part of the free LV inlet, when any enlargement occurs, the posterior mitral annulus has to be displaced toward the LV inlet crest. This results in a decreased posterior leaflet coaptation area, and an increased annulopapillary distance, which tethers the posterior mitral leaflet, alternatively described as atriogenic leaflet tethering [34]. With respect to this hypothesis, Machino-Ohtsuka et al. showed that, in concordance with Silbiger’s theory, in patients with AF who developed any degree of MR and had normal systolic function, the annulus area was significantly larger, as was the tethering angle (angle between the annular plane and the line connecting the annulus and coaptation point) and the nonplanarity angle of the posterior mitral leaflet [27]. Moreover, Ito et al. [35], comparing patients with AFMR, AF without MR, and controls, showed a decreased annular-anterior leaflet coaptation angle in the AFMR cohort, and a larger annular-posterior leaflet tip angle, with the posterior leaflet bent toward the LV. This highlights that the Silbiger hypothesis is echocardiographically evident in some patients; however, the different definitions of the tethering angle may limit the applicability of these findings. Of note, as implied by several investigators, this pathophysiological subtype, despite being clinically identified, is relatively rare and may indicate a more advanced LA remodeling, especially considering the particularly increased LA diameters reported in the aforementioned studies [36].

Based on the aforementioned data, Farhan et al. recently described a pathoanatomic model of AFMR disease progression. In the initial stage, mild LA enlargement may cause slight annulus enlargement, which allows the posterior leaflet to coapt with the anterior [10]. With further dilatation, despite having the posterior leaflet still in the annular plane, the height of coaptation reduces. Further disease progression promotes displacement of the posterior leaflet and further widening of the posterior leaflet angle, obstructing coaptation. Finally, further displacement of the posterior leaflet takes place, causing further height decrease and widening of the angle. In the latest stages, paradoxical motion of the posterobasal LV wall may be noted. This mechanistic description provides a good overview of, mostly, atriogenic leaflet tethering, and AFMR disease progression. However, as noted, AFMR pathogenesis is multifactorial and the cumulative effect of all aforementioned parameters ultimately leads to disease manifestation.

In summary, the pathophysiology of AFMR is complicated and cannot be explained by one sole mechanism. Impaired mitral annular dynamics, secondary to loss of the atrial contraction, direct dilatation promoting malcoaptation, and absence of leaflet enlargement combined result in MR without LV dysfunction. It should be highlighted, however, that some investigators do note that AFMR could not be explained entirely by atrial changes, rather than a combination of ventricular and atrial dysfunction. In specific, Tang et al. [37] studied AFMR, AF, and control patients regarding their atrial and ventricular dynamics. Similar to the aforementioned evidence, the group found significantly decreased presystolic dynamics in AFMR and AF. However, they concomitantly found impaired left ventricular dynamics only in the AFMR cohort. Independent predictors of MR included both atrial (saddle deepening) and ventricular (LV global longitudinal strain). This analysis, thus, supports that, in contrast to this section, atrial mechanics dysregulation cannot alone explain the progression from AF-associated impaired dynamics to clinically overt MR. Given the complexity of the FMR patient, future studies on this context are required, in order to fully understand the distinct AFMR phenotype and its interplay with atrioventricular dynamics.

## 3. AFMR Assessment and Imaging Role

### 3.1. Echocardiographic Findings

Transthoracic (TTE) and transesophageal (TOE) echocardiography are the most common imaging modalities aiding in the diagnosis, identification, and stratification of AFMR, in patients in whom there is high disease suspicion. As described in the pathophysiology of AFMR, it is characterized by normal size of the LV chamber and preserved LV function. Regarding LA, AFMR is characterized by the presence of dilatation of the LA chamber, evaluated by increased LA size and increased LA volume index. The mitral annulus is dilated, its leaflets could be normal or mildly thickened; a pseudo-prolapse of the posterior leaflet could be found [8]. A common characteristic in AFMR is the loss of the normal systolic leaflet concavity toward the LV, which contributes to AFMR being a form of increased leaflet tethering [33]. Regarding the regurgitation’s jet, a central MR jet is commonly found; however, it is not pathognomonic for the disease, as it can also be met in VFMR. An eccentric jet can also be found, but quite often a central jet, due to the adherence to the LA posterior wall (Coanda effect), shares the impression of an eccentric jet, while it is actually a central one, drawn to the adjacent LA wall. Therefore, the direction of the jet is not an element that distinguishes the disease from primary MR or VFMR [38].

Another important aspect that needs to be discussed and evaluated is the quantification of MR severity, which is clinically challenging in FMR and AFMR [36]. Most guidelines suggest the calculation of the vena contracta (VC) and effective regurgitant orifice (ERO) from the proximal isovelocity surface area as the recommended methods for severity estimation (VC ≥ 0.7 cm, ERO ≥ 0.40 cm^2^) [39,40,41]. However, the aforementioned methods are often unsuitable for the estimation of FMR (and AFMR) severity, due to the elliptical regurgitant orifice of the latter. Novel three-dimensional (3D) methods have been suggested, including a 3D estimation of VC and 3D proximal isovelocity surface area, which could surpass this limitation [42]. Another limitation in the AFMR severity quantification, supported by many investigators, is the beat-to-beat variation of cardiac cycle lengths, which in AFMR, due to loss of sinus rhythm, is often impaired [36]. Most guidelines suggest the utilization of an average of at least five heartbeats, which is not easily applicable to a daily routine [43]. It is of great importance to appreciate that there are also limitations of TTE echocardiography, even of 3D echocardiography, toward the evaluation of MV apparatus’s geometry and function [44]. Furthermore, cardiac Computed Tomography (CT) and magnetic resonance (CMR) play a pivotal role, in aiding the multimodality imaging assessment of AFMR [45].

### 3.2. Multimodality Imaging Assessment of AFMR

#### 3.2.1. Cardiac Computed Tomography in AFMR Evaluation

Computed tomography is the imaging modality, mainly utilized as a preprocedural screening tool in transcatheter interventions [10], though when CT is not available, 3D transesophageal echocardiographic (TEE) imaging can provide comparable results [46]. This was supported by a comparative study of imaging modalities (3D-TTE and CT) in 52 patients who were planned for transcatheter MV repair. Coisne et al. demonstrated that both modalities showed excellent correlations, especially regarding the projected mitral annular area and perimeter [47]. The main advantages provided by CT are a comprehensive assessment of MV leaflets’ size and geometry, as well as an elaborated display of both mitral annulus and subvalvular apparatus [10]. Moreover, CT is valuable in ruling out coronary artery disease, as well as identifying calcification of the MV apparatus and accurate anatomic description of the perivalvular area [8]. A rather common setback of both CT and CMR is the necessity of cardiac gating, a fact that in patients with AFMR, in whom often AF coexists, may lead to disturbance in image quality [8].

#### 3.2.2. Cardiac Magnetic Resonance in AFMR Evaluation

It is established knowledge that late gadolinium enhancement CMR is the gold standard for the identification and diagnosis of endocardial fibrous tissue deposition, or in general for the detection of cardiac fibrosis [48]. Myocardial scar, in the presence of severe MR, is highlighted to have a strong correlation with poor prognosis [49]. The multiwoven interplay between AF, LA dilatation, and AFMR has been also described in CMR trials. The DECAAF (Delayed-Enhancement MRI Determinant of Successful Radiofrequency Catheter Ablation of Atrial Fibrillation) trial was a multi-center, prospective, observational cohort that evaluated the relationship between fibrosis, identified by CMR in the atrial myocardium and the recurrence of AF after ablation. Marrouche et al. described that the presence of atrial fibrosis was strongly associated with increased AF recurrence, 1 year after the ablation [50]. Moreover, in another study, this association seemed to extend even 5 years after the procedure [51]. The above findings, given the strong pathogenetic bond between AF and AFMR, suggest that advanced atrial fibrosis could be not only a strong predictor of AF recurrence, but also of the severity of AFMR. Although advanced atrial fibrosis seems to prompt more “resistant-to-ablation” AF, which leads to the maintenance of the structural and functional deleterious effects of AF to the development of AFMR, early fibrosis detection, before the diagnosis of AFMR, could be utilized in clinical trials as an “imaging biomarker” toward this direction [7,52].

Apart from fibrosis, CMR provides us with insightful information regarding the geometry of MV, the in-motion function of mitral apparatus, and the accurate quantification of MR severity, by evaluating all needed hemodynamic parameters. When compared to CT, CMR could be more helpful in the identification of annular distortion or abnormal patterns of leaflet motion annular distortion or abnormal motion of the leaflets [8]. Moreover, quantification of MR severity in CMR has greater reproducibility when compared to echocardiographic measurements [53]. Finally, what distinguishes CMR is not only its ability to accurately measure cardiac chambers’ volumes and flows but the plethora of information (presence of fibrosis, infiltrative disease findings, inflammation, etc.) it provides that aids in the differential diagnosis from other diseases and the confirmation of the type of MR of each patient [54].

#### 3.2.3. Global Longitudinal Strain in AFMR Assessment

Implementation of global longitudinal strain (GLS) has been shown to gain ground in the diagnostic arsenal toward evaluating MR [40]. In primary MR, GLS is a strong predictor of outcomes in surgically treated patients [55], while in patients with severe FMR, GLS has been shown to be significantly associated with LV function, compared to ejection fraction, regarding prognosis of adverse outcomes [56]. In AFMR, the evaluation of the LA strain is a new frontier toward stratifying the diagnosis and prognosis of this entity [57]. It is established knowledge that AFMR, in which disturbance of LA function prevails, is presented with decreased values of left atrium reservoir strain (LARS) [18]. LARS is more associated with LA function, compared to a simple estimation of LA size, in patients with AFMR [58]. In VMFR, reduced LARS (lower than 14%) was associated with a negative prognosis [59]. Matta et al. demonstrated that severe AFMR was associated with impaired both atrial and ventricular strain [60]. Increased LA size and mitral annulus diameter are findings, correlated to decreased values of LARS, in patients with AFMR, prompting the true pathogenetic mechanisms between LA malfunction in AF and the development of AFMR, and suggesting that treating and even preventing the onset of AF might ameliorate the natural history of AFMR [57,60].

## 4. Surgical and Pharmacotherapy Outcomes in AFMR

### 4.1. Current Guidelines Suggestions

Most current guidelines do not differentiate the pharmacological treatment of secondary MR and AFMR, although there is an alternate pathophysiological background. This lack of discrete suggestions is mainly attributed to the scarcity of data, derived from randomized trials, in this population [61]. The 2021 ESC/EACTS Guidelines for the management of valvular heart disease make no specific suggestion regarding this entity [40]. Regarding secondary MR, the 2020 ACC/AHA Guidelines for the Management of Patients with Valvular Heart Disease mention that the best treatment for patients with FMR is not yet clear, as MR is not the cause, but one of the components of the disease [62]. These guidelines suggest that patients with secondary MR in the setting of preserved ejection fraction (LVEF ≥ 50%) should be treated with standard GDMT (Guideline-directed medical therapy) for HF and AF, including ACE inhibitors, ARBs, beta-blockers, aldosterone antagonists, and/or sacubitril/valsartan, and biventricular pacing if indicated (Class IA recommendation). Both European and American guidelines make no specific suggestion for AFMR patients. On the other hand, the 2020 Guidelines of the Japan Circulation Society (JCS) on the Management of Valvular Heart Disease make some unique suggestions as they mention specifically that symptomatic patients with AFMR should be treated with standard heart failure treatment, including diuretics, which may have an impact on reducing LA size (Class IC recommendation), and rhythm control is suggested for symptomatic patients with AFMR and AF (Class IIA recommendation), which may lead to regression of atrial remodeling and restore of atriogenic contribution to dilatation of the atria and of the annulus [41]. Such improvements in normal atrial mechanics and volume following HF treatment are particularly important for patient prognosis, as reductions in left atrial volume have been shown to be predictive of reduced risk for future mortality, HF hospitalization, and heart transplantation in patients with dilated cardiomyopathy, implicating similar benefits in the non-ischemic AFMR phenotype [63].

### 4.2. Sinus Rhythm Restoration and AFMR

Regarding sinus restoration’s impact on LA geometry and function, it is mainly based on the following studies.

Gertz et al. conducted a retrospective study to evaluate the impact of sinus rhythm restoration on MR severity. Toward this aim, they screened 828 patients who underwent the first AF ablation at their center, and from them, 53 patients seemed to present echocardiographic characteristics of at least moderate MR; for the sake of the study, a reference cohort of the same patients’ sample size (53) was selected as control [7]. They demonstrated that MR patients, when compared to control, were older, with clinically persistent AF and larger atria size; furthermore, they showed that patients who underwent successful sinus rhythm restoration at the MR arm were associated with a significant decrease in both left atrial size and annular dimension, as well as lower prevalence of severe MR when compared to the control (24% vs. 82%, *p* = 0.005). Deferm et al. demonstrated in a retrospective analysis of 216 patients with severe MR who underwent mitral valve annuloplasty (97 with AFMR, 119 with VFMR) that 15 patients with AFMR and AF who were treated with electrical cardioversion toward sinus rhythm restoration were associated with a significant reduction in mean EROA from 0.27 to 0.15 cm^2^; moreover, patients with AFMR compared to patients with VFMR were associated with a decreased recurrence of moderate echocardiographic MR echocardiographic (7% vs. 25% at 2 years, overall log-rank *p* = 0.001) and better overall survival rate (adjusted HR 0.43 95% CI 0.22 to 0.82, *p* = 0.011) [64]. Furthermore, AF catheter ablation was associated with better outcomes in this population. In a retrospective study of 136 symptomatic patients with persistent AF and AFMR or VMFR who had undergone AF ablation, Masuda et al. showed that AF ablation was associated with a greater decrease in LA volume and even more importantly with greater prognosis (3.7% vs. 22.6% observation of the composite end-point of all-cause death and HF hospitalization, *p* < 0.0001) when AFMR was present [52]. The above findings were also confirmed in FASTRHAC, a prospective trial in 117 patients hospitalized for AF, demonstrating that mean VC was reduced from 0.40 to 0.21 cm in 47 patients treated with ablation [65]. LARS, as mentioned in the imaging section, seems to be associated with the prediction of AF recurrence, and it is suggested that reduced LARS is a strong predictor of AF recurrence after ablation; therefore, it could be utilized in future trials of AFMR patients in stratifying responders to treatment [66]. Finally, the efficacy of cardioversion is proven to be high in patients with AFMR, as was shown in a retrospective study of 182 patients, undergoing electrical cardioversion; it is noteworthy to add that in patients with AFMR, subclinical inflammation should be treated prior to the cardioversion, as it decreases its efficacy [67].

### 4.3. SGLT2 Inhibitors and Functional MR

Little is known about the impact of long-term SGLT2i on AFMR. However, there are a couple of recently published studies regarding the SGLT2i effect on functional MR and myocardial remodeling. In DEFORM, a prospective, randomized, controlled trial, 104 patients with moderate or severe FMR were enrolled and assigned to either dapagliflozin 10 mg once daily or not, on top of GDMT, and were followed up for 12 weeks [68]. An echocardiographic assessment was performed both at baseline and at follow-up and the primary outcome was the change in EROA; the investigators demonstrated that dapagliflozin, on top of GDMT, was associated with a significant reduction in EROA, regurgitant volume, and improvement in the E/e’ ratio. Furthermore, there were no safety-concerned events in between-group analysis. Although the primary endpoint was not related directly to prognosis (death, myocardial infarction, etc.), DEFORM results suggest that dapagliflozin treatment improves echocardiographic characteristics of patients with FMR and could have an important role in ameliorating cardiovascular prognosis in this population. Recently the results of the EFFORT trial were published. The EFFORT trial (Ertugliflozin for Functional Mitral Regurgitation) was a multi-center, double-blind, RCT evaluating the efficacy of SGLT2i in symptomatic patients with HF (LVEF 35–50%), and functional MR with EROA > 0.1 cm^2^. Toward this aim, 128 patients were enrolled and were assigned to either GDMT (control) or GDMT plus ertugliflozin and were followed up for 12 months. Ertugliflozin, compared to the control arm, was associated with a significant reduction in EROA (−0.05 ± 0.06 vs. 0.03 ± 0.12 cm^2^; *p* < 0.001), RV, LAVI, and LV global longitudinal strain. However, there were not enough data to tell whether the effect of dapagliflozin or ertugliflozin was a “class-effect” in this population; therefore, more RCTs are needed, with better design and even selective enrollment of patients with either AFMR or VFMR to evaluate the distinct effect of SGLT2 inhibition on these populations.

### 4.4. Angiotensin Receptor-Neprilysin Inhibitors and FMR

There is a scarcity of data regarding the effect of angiotensin receptor neprilysin inhibitor (ARNI) for the treatment of AFMR. There is evidence though, regarding its safety and efficacy on patients with FMR. In the PRIME trial, 118 patients with HFrEF and FMR were assigned to either ARNI or valsartan (control) and were followed up for 12 months [69]; Kang et al. demonstrated that the ARNI arm was associated with a significantly greater reduction in EROA, compared to the control, (−0.058 ± 0.095 vs. −0.018 ± 0.105 cm^2^; *p* = 0.032) and with a greater reduction in regurgitant volume (mean difference, −7.3 mL; 95% CI, −12.6 to −1.9; *p* = 0.009), without any between-group differences regarding changes in blood pressure or safety outcomes. In PROVE (Prospective Study of Biomarkers, Symptom Improvement, and Ventricular Remodeling During Sacubitril/Valsartan Therapy for Heart Failure; NCT02887183), a trial with a single-arm, observational design involving treatment with ARNI in patients with HF and at least moderate FMR, ARNI was associated with significant improvement in both clinical and echocardiographic FMR characteristics (decrease in at least moderate MR by 8.2%) [70]. ReReRe is a multi-center, randomized, open-label trial that aims to evaluate the effects of ARNI, compared to valsartan, in LV remodeling and safety outcomes, in patients treated with either ARNI or valsartan after surgery for primary severe AR or MR [71]. Although it is a study mainly on patients with primary MR (or AR), it will provide insightful knowledge regarding the effect of ARNI on this population that could be utilized for the future design of ARNI trials in patients with AFMR.

### 4.5. Mitral Valve Surgery and AFMR

The 2020 Japanese Guidelines make discrete mention of AFMR surgery, as they recommend that surgical intervention should be considered in patients with severe MR who have not responded to GMDT for HF (Class IIa recommendation) [41]. In contrast, the 2020 ACC/AHA Guidelines refer only to FMR and recommend that surgical intervention should be considered in patients with severe MR who have not responded to GMDT for HF, AF, or other comorbidities (Class IIb recommendation) [62]. Both aforementioned recommendations are based on consensus documents (Level of evidence: C), prompting further research into the subject. The 2021 ESC/EACTS Guidelines give no specific suggestions regarding the surgical management of AFMR patients [40]. Restrictive mitral valve annuloplasty is at the core of most surgical procedures in AFMR patients [72,73]. Among other interventions, augmentation of the posterior leaflet with the utilization of a pericardium patch has been studied; however, it was associated with leaflet shrinkage and stiffening, which limits the procedure [74]. Restrictive annuloplasty not only improves annulus geometry, but also leaflet movements, as it was shown that it aids in correcting pseudo-prolapse of the anterior leaflet [75]. Several studies have been performed, investigating the safety and efficacy of MV annuloplasty toward optimizing patients’ outcomes [72].

One of the first trials, assessing MV annuloplasty in 20 patients with AFMR and AF, reported both clinical and echocardiographic improvement, postprocedural; mean LA size was reduced from 6.1 ± 1.6 cm to 5.2 ± 1.0 cm (*p* = 0.03), mean PASP was reduced from 54.1 ± 12.2 mmHg to 40.4 ± 15.5 mmHg (*p* = 0.02), and 85% of the participants were NYHA (New York Heart Association) class I/II at follow-up [76]. In 2015, Takahashi et al., retrospectively evaluating MV annuloplasty in 10 patients with AFMR, demonstrated that MV annuloplasty, accompanied by concomitant tricuspid annuloplasty, was associated with improvement in MR symptoms and reduction in LA size [77]. In another trial in 20 consecutive patients with AFMR, the efficacy and durability of annuloplasty were evaluated in relation to preprocedural echocardiographic findings, showing that MV annuloplasty was associated with a significant decrease in both LAVI (from 94 ± 59 mL/m^2^ to 58 ± 30 mL/m^2^) and mean tricuspid regurgitation peak gradient (from 34 ± 11 mmHg to 23 ± 5 mmHg), respectively, though a larger LV dimension and greater degree of leaflet tethering were associated with an increased rate of MR recurrence [78]. Takahashi et al. evaluated the long-term safety and efficacy (median follow-up of 932 days) of 45 patients with AFMR and permanent AF, undergoing both mitral and tricuspid annuloplasty, reporting that the procedure was both safe and efficient, accompanied by event-free rate of 93%, 87%, and 52% at 1, 3, and 5 years postprocedural, respectively, with the preoperative LAVI being independent predictor of future MACEs [79]. In an observational study, 82 patients with AFMR and permanent AF underwent MV repair, while 52 (63%) underwent concomitant surgical AF ablation; the combined procedure was accompanied by optimal survival rates 97.5% and 92.9% at 1 and 3 years, respectively, while almost all patients (96%) were NYHA I/II at follow-up [80]; remarkably, patients with smaller LA size who received concomitant ablation were associated with better MR recurrence rates [80]. In a small retrospective study of 22 patients who underwent MV surgery, they were divided into two groups, based on whether left atrial plication was simultaneously performed (nine patients). The investigators showed that patients treated both with MV surgery and left atrial plication were associated with a significantly larger reduction in LA size (18.4 ± 7.0 mm vs. 6.9 ± 14.6 mm, *p* = 0.02) and MV angle (16.6 ± 8.1° vs. 1.2 ± 6.9°, *p* < 0.01), compared to MV surgery alone [81].

Minimally invasive endoscopic surgery has been increasingly popular in recent years, associated with similar operating times, less complication rates, and comparable long-term outcomes, without the known disadvantages of conventional sternotomy [82]. Minimally invasive endoscopic MV repair has been also evaluated toward AFMR treatment; for this purpose, Balogh et al. enrolled 131 patients who underwent endoscopic MV repair treatment (MVRepair arm) and 139 patients remaining on the standard of care (GMDT), and they were all followed-up for 5 years, showing that in the MVRepair arm, a total of 88% patients were alive and 80% without readmission for HFpEF at 5 years, proving to be both safe and efficient toward reducing MR symptoms [83]. Moreover, there are studies comparing the efficacy of MV annuloplasty on patients with either atrial or ventricular MR [84,85]. Hirji et al. studied 178 patients with severe MR (94 with AFMR and 84 with VFMR) undergoing MV surgery and compared baseline and survival outcomes between the two groups. They demonstrated that AF was present in 37.2% of the AFMR group and 14.3% of the VFMR group, mean LVEF was 60% and 37%, respectively, and at least moderate LV dilatation was present at 4.8% and 40%, respectively. The operative mortality was 0% in the AFMR cohort vs. 1.2% in the VFMR cohort, while estimates of survival and freedom from reoperation at 5 and 10 years were significantly higher in the AFMR cohort [84]. Carino et al. showed that MV isolated ring annuloplasty in 45 patients with FMR (20 with AFMR and 25 with VFMR) was associated with better MR recurrence rates in the AFMR group (recurrence of MR ≥ 3: 20.8 ± 8.29% vs. 5.9 ± 5.71%) [85]. However, the trial’s setting was retrospective and the sample size was small, prompting the need for larger cohorts with longer follow-up periods.

Lately, there are cohorts with larger than 5 years follow-up period. Kawamoto et al. studied 50 patients, consecutively treated for AFMR with MV surgery, who were followed up for a mean period of 4.6 ± 4.4 years [86]. Of them, 42 patients underwent MV repair with annuloplasty and 8 patients underwent MV replacement. Patients who underwent MV repair did show better long-term outcomes, with a 5- and 10-year freedom from cardiac-related death rate of 93.1% compared to 82.7% in patients who underwent MV replacement. However, the MR recurrence rate was 16.8% for the repair cohort, with partial band annuloplasty, identified in multivariate analysis as an independent predictor of MR recurrence, highlighting that partial band annuloplasty, compared to complete MV ring annuloplasty, should not be recommended regarding MR recurrence [86,87].

Recently, there has been some data regarding the combined MV annuloplasty and Cox-maze procedure in this population; in a prospective cohort of 247 patients with AFMR, undergoing both procedures, the investigators demonstrated that MV repair combined with the Cox-maze procedure was safe and feasible, while patients with initially LVEF ≥ 50% were associated with better long-term MR recurrence rate, compared to those with LVEF of 40–50% [88]. Finally, in a propensity score analysis trial of patients with MR of all origins, undergoing MV repair/replacement, 164 patients were classified as degenerative MR and 82 patients as AFMR. The MR recurrence rate of significant MR after repair did not significantly differ while 5-year freedom from MR recurrence (≥moderate) was 89.8% and 93.0%, respectively [89].

## 5. Transcatheter Edge-to-Edge Repair (TEER) Outcomes in AFMR

Transcatheter Edge-to-Edge Repair (TEER) is a relatively novel alternative to surgery for managing severe MR. Several randomized trials in recent years have documented the safety and efficacy of mitral TEER in symptomatic patients with severe MR, mostly in functional [90,91,92], but also in degenerative disease [93,94]. Given the favorable results of these studies, European Society of Cardiology guidelines on valvular heart disease recommend mitral TEER as an option for managing secondary MR with a class II recommendation in patients with a marked decline in their functional capacity, despite under optimal, maximum tolerated guideline-directed pharmacotherapy [40]. For primary MR, the suggestion still exists, however, only in patients with inoperable or very high surgical risk and echocardiographic suitable anatomy. Similar recommendations are made by the respective American guidelines [62]. However, given the recent recognition of the AFMR phenotype, there are no specific recommendations for this type of patient, nor does a consensus document exist to guide clinical decision making. It is noteworthy that there is a lack of randomized data on the safety and efficacy of TEER in the AFMR phenotype; however, a number of observational studies have tried to address the question of whether treating this cohort of individuals with TEER has comparable outcomes to the ordinary intervention indications (Table 1).

Single-arm studies have evaluated TEER in the context of AFMR. Tanaka et al. [95] evaluated 118 patients with AFMR undergoing TEER for severe MR, with a mean age of 80 ± 8 years and 39.8% being male. The technical success rate of the procedure was 94.1%, while MR ≤ 1+ was achieved in 79.7% of patients. The reported in-hospital mortality was 2.5%. Regarding predictors of MR reduction, the study found significantly less incidence of MR reduction in patients with larger left atrial volume index or low leaflet-to-annulus index, and increased reduction in patients receiving newer generation MitraClip systems.

More extensive follow-up was provided by the study of Rubbio et al. [96], which followed up the 87 patients included in the study for 2 years. Similar to the previous short-term results were reported, with a 5% all-cause mortality rate at 30 days. At 2 years, the estimated freedom from all-cause and cardiovascular mortality was 60% and 77%, respectively, while freedom from all-cause mortality and HF hospitalization was 55%. Predictors of all-cause mortality were MR ≥ 2+ (HR 5.40; 95%CI 1.37–21.27) and inter-commissural annular diameter ≥ 35 mm (HR 4.16; 95%CI: 1.06–16.36).

### 5.1. AFMR and VFMR Comparison

Aiming to investigate the efficacy of the procedure in contrast to the VFMR phenotype, several investigators performed comparative analyses of patient and procedural outcomes after TEER in VFMR and AFMR.

Claeys et al. [97] aimed to assess the clinical effect and hemodynamic impact of TEER in AFMR, compared to VFMR. Thus, they compared 52 AFMR patients with 307 VFMR patients. At 6 months follow-up, the reduction in MR was not different and non-inferior in AFMR, compared with VFMR (MR grade ≤ 2 in 94% vs. 82%, respectively, *p* < 0.001 for noninferiority). Furthermore, both cohorts had meaningful improvement in the NYHA class, without a significant difference between groups. Regarding MACEs, these were significantly lower in the AFMR group (adjusted OR: 0.46; 95%CI: 0.24–0.88), mostly driven by the reduction in HF rehospitalization. In respect to hemodynamics, there was a more pronounced reduction in the systolic pulmonary artery pressure at rest in the AFMR cohort, rather than the VFMR (*p* = 0.03).

Similar results were provided by Benito-Gonzalez et al. [98], who analyzed the Spanish Mitraclip Registry, evaluating individuals with AFMR. Of the total FMR cohort, 4.5% (45 patients) were classified as AFMR. The procedural success rate was 91.7%, with survival free from HF readmission being close to 75%. Post-procedural MR < 1+ was achieved in 74.5%, with a mean mitral valve gradient (MVG) of 3.08 ± 1.25 mmHg. Significant improvement was noted in the NYHA class from baseline (NYHA III-IV: 89.6%) to 6 months and 1-year follow-up (NYHA III-IV: 21.7%). No statistically significant difference was found between VFMR and AFMR in the aforementioned parameters.

More extensive follow-up becomes available from analyses of the EXPAND registry, where 53 patients with AFR and 360 with VFMR undergoing TEER were followed up for 1 year [105]. There was a similar reduction in MR severity in both groups at 1 year (MR ≤ 2; AFMR: 100%, VFMR: 99.5%), while there were significant improvements in the NYHA class and Kansas City Cardiomyopathy Questionnaire score from baseline in both groups. Notably, at the 1-year follow-up, adverse events related to the valve leaflets were not frequent in both cohorts (1.9% in both cohorts). Sustained reductions in MR severity and NYHA class were also noted by Doldi et al. in the EuroSMR registry, with no difference in the 2-year survival between AFMR, non-AFMR, and VFMR phenotypes, with an estimated AFMR 2-year survival rate at 70.4% [99].

Furthermore, a recent study by Tanaka et al. [100] enrolled 125 patients with AFMR, out of 441 FMR patients. They included both MitraClip (92.0%) and PASCAL (8.0%) systems. In terms of procedural outcomes, residual MR ≤ 1+ rate was comparable between AFMR and VFMR patients (76.8% vs. 72.2%; *p* = 0.27), while an MPG ≥ 5 mmHg was more frequent in patients with AFMR (21.6% vs. 13.3%; *p* = 0.030). Regarding adverse outcomes at 1-year follow-up, the investigators note that in both cohorts, a residual MR grade ≤ 1+ was linked to a lower risk of death and HF hospitalization. MPG ≥ 5 mmHg was also associated with adverse outcomes, but only in the AFMR cohort. In particular, the adverse event rates for the MR ≤ 1+ and an MPG ≥ 5 mmHg phenotype was 50.7%, for those with MR > 1+ 41.8% and for MR ≤ 1+ and an MPG < 5 mmHg 14.3%, highlighting the importance of optimal MR reduction.

### 5.2. AFMR, VFMR, and DMR Comparison

Investigators aimed to compare TEER outcomes in AFMR with both VFMR and DMR phenotypes. In this context, Yoon et al. [101] included in their study, 116 AFMR patients, 505 VFMR patients, and 423 DMR patients undergoing TEER. Regarding baseline characteristics, AFMR patients were more commonly women and had diagnosed atrial fibrillation, as well as increased rates of tricuspid regurgitation and larger left and right atria, whereas patients with VFMR were more commonly younger, but with higher STS scores and lower LVEF. Regarding technical success, MR severity more than moderate at the time of discharge was not different between the three arms (AFMR: 5.2%; VFMR: 3.2%; DMR 2.6%; *p* = 0.37). However, the composite of all-cause mortality and HF rehospitalization at 2 years was higher in patients with FMR compared to DMR (AFMR: 31.5%, VFMR: 42.3%, and DMR: 21.6%), with significantly lower rates in AFMR rather than VFMR (HR: 0.69; 95%CI: 0.50–0.95; *p* = 0.022).

In a similar manner, Simard et al. [102] showed that patients with AFMR had no difference from VFMR and DMR in achieving device success; however, patients with AFMR were less likely to achieve a great (≥3 grades) MR reduction compared to both pathologies (19% vs. 54%; *p* = 0.01 and 49.7%; *p* = 0.01). With respect to left atrial dynamics, only patients with VFMR and DMR experienced a significant reduction in mean LA pressure and peak LA v-wave, with other echocardiographic outcomes being similar between groups.

Finally, Masiero et al. [103] also compared the outcomes of VFMR and AFMR, but discriminated the etiology of VFMR into ischemic (iVFMR) and non-ischemic (niVFMR). Out of the total 1153 patients, 6% had aFMR, 47% iVFMR, and 47% niVFMR. The investigators reported similar procedural success in all groups. Importantly, at 2 years follow-up, the AFMR cohort had lower cardiovascular mortality and HF rate than both iVFMR (Hazard Ratio (HR): 0.43, *p* = 0.02) and niVFMR (HR:0.45; *p* = 0.03). In particular, the trialists report that, along with postprocedural MR grade greater than 1+ and peripheral vasculopathy, the non-AFMR phenotype was an independent predictor of adverse outcomes.

### 5.3. Disproportionate AFMR

In the context of disproportionate MR, i.e., higher MR grade in relation to LV size, which has a well-established effect on TEER outcomes, Doldi et al. [104] recently explored whether the same principle applies to patients with AFMR, and if it can alter patient outcomes. In specific, employing the EROA/LVEDV ratio to differentiate proportionate from disproportionate AFRM, 98 patients were included and followed up for 2 years. Procedural success was similar between the two arms; however, patients with disproportionate MR had numerically higher systolic PAP and NYHA class > III. Furthermore, disproportionate AFMR was significantly linked to increased 2-year mortality (*p* < 0.001), with the EROA/LVEDV ratio and tricuspid annular plane systolic excursion (TAPSE) being the two identified predictors of 2-year mortality.

### 5.4. Data from Meta-Analyses

Given the paucity of randomized data and the several observational studies, Hamada et al. [106] performed a meta-analysis of the safety and efficacy of TEER in AFMR. A total of eight studies were included. MR grade ≤ 2 was reported in 93.7% in the short (0–6 months) and 97.1% in the long (2–24 months) term follow-up. No difference in postprocedural MR grade ≤ 2, NYHA class ≤ 2, and all-cause mortality was identified between VFMR and AFMR in both follow-ups, resulting that TEER has a similar clinical efficacy and safety profile in AFMR and VFMR. Similar conclusions were also drawn by another meta-analysis from Moras et al. [107].

Available, to date, studies and meta-analyses confirm that TEER in patients with AFMR has comparable, or even better, outcomes, compared to VFMR. However, the lack of randomized trials in this setting is noticeable, and therefore, safe conclusions cannot yet be extracted. Nevertheless, the aforementioned observational studies indicate a signal toward the benefit of TEER in the AFMR subtype, with equal to VFMR safety and efficacy. The potential benefit over VFMR in this cohort may result from the less high-risk clinical profile of these patients, as well as from the increased technical success noted in some studies. Furthermore, the effects of TEER in the annular and LA dynamics may be responsible for the observed benefit. Whether TEER is the optimal transcatheter option for AFMR, as well as the reasons behind a potential benefit over the VFMR phenotype, is still to be investigated in future trials, where more distinct anatomic criteria of suitability for TEER will have to be defined, similarly to those for VFMR, as well as comparisons with other percutaneous repair techniques.

## 6. Clinical Perspectives

**Figure 1:**
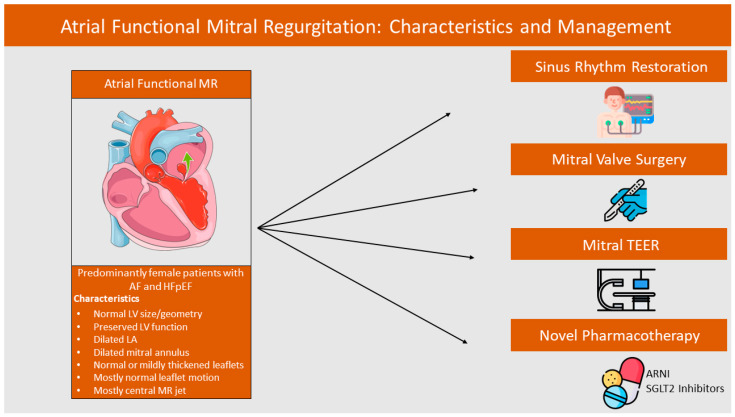
Atrial Functional Mitral Regurgitation: Characteristics and Management. Abbreviations: AF: Atrial Fibrillation; HFpEF: Heart Failure with Preserved Ejection Fraction; LV: Left Ventricle; LA: Left Atrium; MR: Mitral Regurgitation; TEER: Transcatheter Edge-to-Edge Repair; ARNI: Angiotensin Receptor-Neprilysin Inhibitor; SGLT2: Sodium Glucose Co-Transporter 2.

As analyzed, the increasingly recognized phenotype of AFMR poses a diagnostic and therapeutic challenge, which is now being more actively researched, aiming to identify the optimal diagnostic tools, prognostic markers, and therapeutic interventions (Figure 1). AFMR is not a condition but rather a phenotype of many simultaneous pathophysiological cascades. Therefore, the need for an individualized evaluation and assessment of each patient is of great importance. The concomitant diseases have a complex, multifactorial role in the natural history of AFMR. Thus, the identification and treatment of each plays a pivotal part, and there is an urgent need for further evaluation of both novel pharmacotherapy treatment and RCTs, comparing medical treatment to surgery and to TEER in patients with severe MR. Finally, little is known about the need for urgent treatment of this entity and further research is required [108].

TEER has shown safety and efficacy in patients with AFMR in the aforementioned analyzed studies. However, the majority of the studies have been conducted with the use of a MitraClip device, and not with other TEER devices, such as Pascal. A recent case report by Liu et al. [109] showed the effect of the DragonFly system on a patient with AFMR. The DragonFly system is a leaflet coaptation device consisting of a central nitinol spacer that coapts the leaflets without tensioning them. In this report, post-interventionally, the patient had a reduction in MR by 4+ to 1+, with a mean inflow gradient of 4 mm, and no adverse events were noted in the post-procedural or follow-up period. More extensive evaluation of other leaflet coaptation devices is, thus, necessary in the future. Furthermore, there are questions regarding whether a predominantly annular pathology should be treated with leaflet approximation, rather than percutaneous annuloplasty. In this context, Rottländer et al. [110] described the use of the Carillon Mitral Contour system, a percutaneous coronary sinus-based mitral annuloplasty device, in AFMR and VFMR patients. Following device placement and post-interventional echo, mitral annuloplasty had a comparable reduction in VC, ERO, and regurgitant volume in both pathologies, while also showing enhanced MR classification post-interventionally and at 3-months follow-up. A following analysis [111], comparing MitraClip with Carillon in AFMR patients, had 100% procedural success in both arms, with comparable functional improvement at 3 and 12 months follow-up. However, the quantitative echocardiographic reduction in MR was more pronounced after TEER, as was the MR severity classification, without differences in qualitative parameters. Recognizing the limitations of retrospective studies and given the positive early results of annuloplasty, more research is ongoing with Carillon and other annuloplasty devices, in order to determine their role in AFMR patient management.

It is important to note that in patients with AFMR, tricuspid regurgitation (TR) may be of important significance, with some investigators calling it bilateral atrioventricular valve disease or dual-valve disease [75], while treating both AFMR and atrial functional TR (AFTR) in surgical studies led to improved survival and less HF admissions [79]. AFTR is recognized by the absence of right ventricular dysfunction, and a predominant annulus dilatation, which may regard 10–15% of all clinically relevant TR [112]. Pathophysiologically, AF and right atrial dilatation, resulting in tricuspid annulus dilatation and annulus/leaflet imbalance, may also be considered the main culprits. A recent definition for AFTR, provided by Muraru et al. indicates that the criteria that should be fulfilled are the following: clinically relevant FTR, predominant annulus dilatation, predominant right atrium dilatation with increased end-systolic right atrial/ventricular ratio, absence of significant tricuspid leaflet tethering, right ventricle conical remodeling, and preserved systolic function of both ventricles [112]. Prognostically, patients with AFTR seem to have better survival (10-year survival 78%) compared to VFTR (10-year survival 46%, *p* < 0.001), independently of clinical and echocardiographic characteristics [113]. Regarding tricuspid TEER, Schlotter et al. [114] defined AFTR as a tenting height ≤ 10 mm, midventricular right ventricle diameter ≤ 38 mm, and LVEF ≥ 50%, and showed significantly better long-term survival than non-AFTR in both conservatively treated (*p* < 0.01) and tricuspid valve repair cohorts (*p* < 0.05). Given the current anatomical considerations for a successful tricuspid TEER procedure (decreased coaptation gap and tethering) [115], more research is needed in order to identify whether tricuspid TEER is an effective therapy for AFTR. Nevertheless, given the significance in the management of AFMR as well, this new research frontier will provide in the near future more answers to questions involving optimal patient selection, optimal device, and timing, especially considering the co-existence and treatment of AFMR.

## 7. Conclusions

AFMR is mainly attributed to altered structural and functional mechanics of the left atrium and the mitral valve apparatus, in the presence of AF or HFpEF. Novel imaging techniques, such as left atrial strain and late-enhancement CMR, have been shown to be associated with early disease detection and prognosis. Multimodality imaging will aid in the proper establishment of diagnostic criteria in future trials, as well as in individualized decision making in clinical practice. Finally, despite the promising results, there is a large need for randomized trials to compare the safety and efficacy of surgery vs. TEER in the setting of severe disease.

## Figures and Tables

**Table 1:** Studies assessing TEER in Atrial Functional MR.

Study, Year	Participants (n)	Mean Age (years)	Gender (Female)	AF	Device Type	Post-Procedure Echocardiographic Assessment			Outcomes				
						Technical Success	MR < 2+	MR < 1+	NYHA Class I/II	CV Mortality	All-Cause Mortality	HF	Adverse Events
Tanaka et al. [95], 2022	118 AFMR patients	80 ± 8	60.2%	90.7%	MitraClip (all generations)	94.1%	90.7%	79.7%	NR	0%	2.5%	NR	None
Rubbio et al. [96], 2022	87 AFMR patients	81 (78–83)	61%	100%	MitraClip (all generations)	97%	89%	NR	79%	4%	5%	4%	AKI:8% Major Bleeding: 4.5%
Claeys et al. [97], 2021	52 AFMR and 307 V-FMR patients	79 ± 8 vs. 72 ±10	54% vs. 28%	63% vs. 49%	MitraClip	92% vs. 92%	94% vs. 82% (p_non-inferiority_ < 0.001)	NR	90% vs. 80% (*p* = NS)	NR	0% vs. 3.3% (*p* = NS)	95% vs. 87%	No significant difference
Benito-Gonzalez et al. [98], 2021	48 AFMR patients vs. 624 VFMR patients	78.0 ± 7.5 vs. 70.7 ± 10.4	52.1% vs. 24%	100% vs. 56.1%	MitraClip	97.9% vs. 96% (*p* = NS)	94.6% vs. 95.3%	74.5% vs. 61.7%	78.2% vs. 74.7%	AFMR: 74.9% free of events			No difference
Doldi et al. [99], 2022	126 AFMR patients vs. 1349 VFMR patients vs. 133 non-AFMR	80.3 (76.3–83.4) vs. 74.0 (68.0–79.0) vs. 78.5 (74.6–82.0)	61.1% vs. 30.2% vs. 50.4%	78.6% vs. 58.2% vs. 64.7%	MitraClip	87.2% vs. 94% vs. 93.3%	87.2% vs. 94% vs. 93.3%	NR	AFMR: 73.4%	No difference between groups			
Tanaka et al. [100], 2023	125 AFMR patients vs. 316 VFMR patients	80 ± 6 vs. 76 ± 8	66.8% vs. 38.0%	92.8% vs. 73.1%	Mitraclip (92%), PASCAL: 8%	NR	92.0% vs. 93.9%	77.6% vs. 71.2%	NR	AFMR: Residual MR with an MPG ≥ 5 mmHg was associated with a higher risk of the composite outcome (HR 2.31, 95% CI: 1.11–4.83; *p* = 0.025) (VFMR: Residual MR ≤ 1+ was also associated with a lower risk of the composite outcome (HR 0.56, 95% CI: 0.35–0.88; *p* = 0.012);			
Yoon et al. [101], 2022	116 AFMR patients vs. 505 VFMR patients vs. 423 DMR patients	78.8 ± 9.7 vs. 72.1 ± 12.8 vs. 80.6 ± 10.5	56.0% vs. 38.0% vs. 40.4%	72.4% vs. 52.5% vs. 52.2%	MitraClip	NR	94.8% vs. 96.8% vs. 97.4%	86.2% vs. 84.7% vs. 86.0%	NR	VFMR vs. AFMR vs. DMR at 2 years: 31.1% vs. 26.8% vs. 17.7%, respectively		NR	NR
Simard et al. [102], 2022	21 AFMR patients vs. 37 VFMR patients vs. 191 DMR patients	82.4 ± 5.9 vs. 74 ± 9.4 vs. 79.8 ± 12.7	23.8% vs. 21.6% vs. 39.3%	76.2% vs. 67.6% vs. 63.9%	MitraClip	NR	90.4% vs. 89.1% vs. 90.6%	52.3% vs. 64.8% vs. 67%	63.6% vs. 51.9% vs. 67.5%	NR	14% vs. 27% vs. 18%	NR	MACE at 1 year: 48% vs. 46% vs. 32%
Masiero et al. [103], 2024	71 AFMR patients vs. 541 iFMR patients vs. 541 niFMR patients	79 [75–84] vs. 73 [69–79] vs. 72 [67–79]	58% vs. 21% vs. 35%	76% vs. 43% vs. 54%	MitraClip (all generations)	100% vs. 98% vs. 99%	97.2% vs. 91.1% vs. 90.4%	67.6% vs. 57.5% vs. 54%	80% vs. 79% vs. 90%	3.1% vs. 2.4% vs. 2.7%	4.8% vs. 3.2% vs. 4.4%	1.7% vs. 3.1% vs. 2.8%	No difference in periprocedural adverse events
Doldi et al. [104], 2024	98 patients with AFMR (77 included, 52 proportionate, 25 disproportionate)	80.0 [76.1, 84.0] vs. 81.0 [77.5, 83.5]	65.4% vs. 76%	76.9% vs. 84%	MitraClip	NR	Similar between groups	84.3% vs. 80%	NR	NR	Disproportionate MR was associated with increased 2-year mortality (56% vs. 68%; *p* < 0.001)		NR

## Data Availability

Not applicable.
